# Supplementary material for: Extended graphical lasso for multiple interaction networks for high dimensional omics data
Source: PLoS Comput Biol. 2021 Oct 20;17(10):e1008794. doi: 10.1371/journal.pcbi.1008794 (PMC8528283; doi:10.1371/journal.pcbi.1008794)
Supplement: S1 Text — (PDF) [file pcbi.1008794.s001.pdf]

S1 Text: A detailed ADMM for the extended joint hub graphical lasso

Since the objective function is completely separable with respect to the variables $(\Theta^{(k)}, Z^{(k)}, V^{(k)})$, updating X_{t+1} can be achieved by updating each variable.

$$\Theta_{t+1}^{(k)} = \operatorname{argmin}\left\{-n_k(\log(\det(\Theta^{(k)})) - \operatorname{tr}(S^{(k)}\Theta^{(k)})) + \frac{\rho}{2}||\Theta^{(k)} - \tilde{\Theta}_t^{(k)} + W_{\Theta_t}^{(k)}||_F^2\right\}. \tag{1}$$

Let UDU^T denote the eigen decomposition of $S^{(k)} - \frac{\rho}{n_k}(\tilde{\Theta}_t^{(k)} - W_{\Theta_t}^{(k)})$, the solution is given by $U\hat{D}U^T$, where $\hat{D}$ is the diagonal matrix with j^{th} diagonal element as follows:

$$\frac{n_k}{2\rho}(-D_{jj} + \sqrt{D_{jj}^2 + \frac{4\rho}{n_k}})$$

$$\begin{aligned} Z_{t+1} = \operatorname{argmin}\Bigg\{\lambda_1 \sum_{k=1}^{K} ||Z^{(k)} - diag(Z^{(k)})||_1 + \lambda_2 \sum_{k<k'} ||Z^{(k)} - Z^{(k')} \\ - diag(Z^{(k)} - Z^{(k')})||_1 + \sum_{k=1}^{K} \frac{\rho}{2}||Z^{(k)} - \tilde{Z}_t^{(k)} + W_{Z_t}^{(k)}||_F^2\Bigg\}. \end{aligned} \tag{2}$$

The objection function is completely separable with respect to each pair of matrix elements $\{(i,j), i \neq j\}$. Therefore, it is equivalent to solve separate optimization problems.

$$\begin{aligned} Z_{tij} = \operatorname{argmin}\Bigg\{\lambda_1 \sum_{k=1}^{K} |Z_{ij}^{(k)}| + \lambda_2 \sum_{k<k'} |Z_{ij}^{(k)} - Z_{ij}^{(k')}| \\ + \sum_{k=1}^{K} \frac{\rho}{2}(Z_{ij}^{(k)} - \tilde{Z}_{tij}^{(k)} + W_{Z(tij)}^{(k)})^2\Bigg\}. \end{aligned} \tag{3}$$

This form belongs to a class of fused lasso problems[2].

$$\begin{aligned} V_{t+1}^{(k)} = \operatorname{argmin}\Bigg\{\lambda_3||V^{(k)} - diag(V^{(k)})||_1 + \lambda_4||V^{(k)} - diag(V^{(k)})||_{1,2} \\ + \frac{\rho}{2}||V^{(k)} - \tilde{V}_t^{(k)} + W_{V_t}^{(k)}||_F^2 + \frac{\rho}{2}||\tilde{\tilde{V}}_t^{(k)} - V^{(k)} + \tilde{W}_{V_t}^{(k)}||_F^2\Bigg\}, \end{aligned} \tag{4}$$

the objective function is column separable. Let $(V_{t+1})_j$ be the jth column of matrix V_{t+1}, we solve

$$\begin{aligned}(V_{t+1})_j^{(k)} &= \text{argmin}\Big\{\lambda_3||V_j^{(k)}||_1 + \lambda_4||V_j^{(k)}||_2 + \frac{\rho}{2}||V_j^{(k)} - (\tilde{V}_t)_j^{(k)} + (W_{V_t})_j^{(k)}||_2^2 \\ &\quad + \frac{\rho}{2}||(\tilde{\tilde{V}}_t)_j^{(k)} - V_j^{(k)} + (\tilde{W}_{V_t})_j^{(k)}||_2^2\Big\} \\ &= \text{argmin}\Big\{\lambda_3||V_j^{(k)}||_1 + \lambda_4||V_j^{(k)}||_2 - \rho < V_j^{(k)}, (\tilde{V}_t)_j^{(k)} + (\tilde{\tilde{V}}_t)_j^{(k)} - (W_{V_t})_j^{(k)} + (\tilde{W}_{V_t})_j^{(k)} > \\ &\quad + \rho||V_j^{(k)}||_2^2 + \frac{\rho}{2}||(\tilde{V}_t)_j^{(k)} - (W_{V_t})_j^{(k)}||_2^2 + \frac{\rho}{2}||(\tilde{\tilde{V}}_t)_j^{(k)} + (\tilde{W}_{V_t})_j^{(k)}||_2^2\Big\}.\end{aligned} \tag{5}$$

It is equivalent to solve the optimization problem

$$(V_{t+1})_j^{(k)} = \text{argmin}\Big\{\lambda_3||V_j^{(k)}||_1+\lambda_4||V_j^{(k)}||_2+\rho||V_j^{(k)}-((\tilde{V}_t)_j^{(k)}+(\tilde{\tilde{V}}_t)_j^{(k)}-(W_{V_t})_j^{(k)}+(\tilde{W}_{V_t})_j^{(k)})/2||_2^2\Big\}. \tag{6}$$

The result is given in [1].

$$\tilde{\tilde{V}}_{t+1} = \text{argmin}\Big\{\lambda_5 \sum_{k<k'} ||\tilde{\tilde{V}}^{(k)}-\tilde{\tilde{V}}^{(k')}-diag(\tilde{\tilde{V}}^{(k)}-\tilde{\tilde{V}}^{(k')})||_1+\frac{\rho}{2}\sum_{k=1}^{K}||\tilde{\tilde{V}}^{(k)}-V_{t+1}^{(k)}+\tilde{W}_{V_t}^{(k)}||_F^2\Big\}, \tag{7}$$

it is similar to the solution for Z.

Although the objective function is not separable with respect to the variables $(\tilde{\Theta}^{(k)}, \tilde{Z}^{(k)}, \tilde{V}^{(k)})$, updating $\tilde{X}_{t+1}$, can be achieved as follows:

$$\Pi_{t+1}^{(k)} = \frac{\rho}{6}\Big(\Theta_{t+1}^{(k)} + W_{\Theta_t}^{(k)} - Z_{t+1}^{(k)} - W_{Z_t}^{(k)} - V_{t+1}^{(k)} - W_{V_t}^{(k)} - t(V_{t+1}^{(k)} + W_{V_t}^{(k)})\Big) \tag{8}$$

$$\tilde{\Theta}^{(k)} = -\frac{\Pi_{t+1}^{(k)}}{\rho} + \Theta_{t+1}^{(k)} + W_{\Theta_t}^{(k)} \tag{9}$$

$$\tilde{Z}^{(k)} = \frac{\Pi_{t+1}^{(k)}}{\rho} + Z_{t+1}^{(k)} + W_{Z_t}^{(k)} \tag{10}$$

$$\tilde{V}^{(k)} = 2\frac{\Pi_{t+1}^{(k)}}{\rho} + V_{t+1}^{(k)} + W_{V_t}^{(k)} \tag{11}$$

Derivation of (8)

$$
\begin{aligned}
\tilde{X}_{t+1} &= \operatorname{argmin}_{\tilde{X}} \left\{ \Psi(\tilde{X}) + \frac{\rho}{2}||X_{t+1} - \tilde{X} + W_t||_F^2 \right\} \\
&= \operatorname{argmin} \left\{ \sum_{k=1}^{K} I\big(\tilde{\Theta}^{(k)} = \tilde{Z}^{(k)} + \tilde{V}^{(k)} + t(\tilde{V}^{(k)})\big) + \frac{\rho}{2}||X_{t+1} - \tilde{X} + W_t||_F^2 \right\} \\
&= \operatorname{argmin} \left\{ \frac{\rho}{2} \sum_{k=1}^{K} ||\tilde{\Theta}^{(k)} - (\Theta_{t+1}^{(k)} + W_{\Theta_t}^{(k)})||_F^2 \right. \\
&\quad \left. + \frac{\rho}{2} \sum_{k=1}^{K} ||\tilde{Z}^{(k)} - (Z_{t+1}^{(k)} + W_{Z_t}^{(k)})||_F^2 + \frac{\rho}{2} \sum_{k=1}^{K} ||\tilde{V}^{(k)} - (V_{t+1}^{(k)} + W_{V_t}^{(k)})||_F^2 \right\},
\end{aligned}
$$

where $\tilde{\Theta}^{(k)} = \tilde{Z}^{(k)} + \tilde{V}^{(k)} + t(\tilde{V}^{(k)})$.

The Lagrange form of the above formula is

$$
\begin{aligned}
& L(\tilde{\Theta}^{(k)}, \tilde{Z}^{(k)}, \tilde{V}^{(k)}, \Pi^{(k)}) \\
= & \frac{\rho}{2} \sum_{k=1}^{K} ||\tilde{\Theta}^{(k)} - (\Theta_{t+1}^{(k)} + W_{\Theta_t}^{(k)})||_F^2 + \frac{\rho}{2} \sum_{k=1}^{K} ||\tilde{Z}^{(k)} - (Z_{t+1}^{(k)} + W_{Z_t}^{(k)})||_F^2 \\
& + \frac{\rho}{2} \sum_{k=1}^{K} ||\tilde{V}^{(k)} - (V_{t+1}^{(k)} + W_{V_t}^{(k)})||_F^2 + \sum_{k=1}^{K} \operatorname{tr}\Big(\Pi^{(k)} \big(\tilde{\Theta}^{(k)} - (\tilde{Z}^{(k)} + \tilde{V}^{(k)} + t(\tilde{V}^{(k)})) \big) \Big)
\end{aligned}
$$

$$
\begin{aligned}
\frac{\partial L}{\partial \tilde{\Theta}^{(k)}} &= \rho\big(\tilde{\Theta}^{(k)} - (\Theta_{t+1}^{(k)} + W_{\Theta_t}^{(k)})\big) + \Pi^{(k)} = 0 \\
\frac{\partial L}{\partial \tilde{Z}^{(k)}} &= \rho\big(\tilde{Z}^{(k)} - (Z_{t+1}^{(k)} + W_{Z_t}^{(k)})\big) - \Pi^{(k)} = 0 \\
\frac{\partial L}{\partial \tilde{V}^{(k)}} &= \rho\big(\tilde{V}^{(k)} - (V_{t+1}^{(k)} + W_{V_t}^{(k)})\big) - 2\Pi^{(k)} = 0 \\
\frac{\partial L}{\partial t(\tilde{V}^{(k)})} &= \rho\big(t(\tilde{V}^{(k)}) - t(V_{t+1}^{(k)} + W_{V_t}^{(k)})\big) - 2\Pi^{(k)} = 0
\end{aligned}
$$

For each k, the first equality subtracts the last three equalities and we get

$$
\rho\big(Z_{t+1}^{(k)} + W_{Z_t}^{(k)} + V_{t+1}^{(k)} + W_{V_t}^{(k)} + t(V_{t+1}^{(k)} + W_{V_t}^{(k)}) - (\Theta_{t+1}^{(k)} + W_{\Theta_t}^{(k)})\big) + 6\Pi^{(k)} = 0
$$

Therefore

$$
\Pi_{t+1}^{(k)} = \frac{\rho}{6}\Big((\Theta_{t+1}^{(k)} + W_{\Theta_t}^{(k)}) - (Z_{t+1}^{(k)} + W_{Z_t}^{(k)}) - (V_{t+1}^{(k)} + W_{V_t}^{(k)}) - t(V_{t+1}^{(k)} + W_{V_t}^{(k)}) \Big)
$$

Reference

[1] Patrick Danaher, Pei Wang, and Daniela M Witten. The joint graphical lasso for inverse covariance estimation across multiple classes. *Journal of the Royal Statistical Society: Series B (Statistical Methodology)*, 76(2):373–397, 2014.

[2] Holger Hoefling. A path algorithm for the fused lasso signal approximator. *Journal of Computational and Graphical Statistics*, 19(4):984–1006, 2010.
