# Supplementary material for: Extended graphical lasso for multiple interaction networks for high dimensional omics data
Source: PLoS Comput Biol. 2021 Oct 20;17(10):e1008794. doi: 10.1371/journal.pcbi.1008794 (PMC8528283; doi:10.1371/journal.pcbi.1008794)
Supplement: S2 Text — (PDF) [file pcbi.1008794.s002.pdf]

S2 Text: The proof of the convergence of the ADMM algorithm for EDOHA

The scaled augmented Lagrangian can be rewritten as

$$L_\rho(X,\tilde{X},\tilde{\tilde{V}},U,\tilde{U}_V)=\Phi(X)+h_1(\tilde{\tilde{V}})+\Psi(\tilde{X})+U^T(X-\tilde{X})+\frac{\rho}{2}\|X-\tilde{X}\|_F^2 \\ +\tilde{U}_V^T(\tilde{\tilde{V}}-V)+\frac{\rho}{2}\|\tilde{\tilde{V}}-V\|_F^2 \tag{1}$$

where $U=\rho W$ and $\tilde{U}=\rho\tilde{W}_V$. Let $(X^*,\tilde{X}^*,\tilde{\tilde{V}}^*)$ be a primal optimal and (U^*,U_V^*) be a dual optimal point. According to convex optimization theory, KKT conditions guarantee that the optimal duality gap is zero [1], that is,

$$L_0(X^*,\tilde{X}^*,\tilde{\tilde{V}}^*,U,\tilde{U}_V)\le L_0(X^*,\tilde{X}^*,\tilde{\tilde{V}}^*,U^*,\tilde{U}_V^*)\le L_0(X,\tilde{X},\tilde{\tilde{V}},U^*,\tilde{U}_V^*)$$

holds for all $X,\tilde{X},\tilde{\tilde{V}},U,\tilde{U}_V$. Since $(X^*,\tilde{X}^*,\tilde{\tilde{V}}^*,U^*,\tilde{U}_V^*)$ is a saddle point for L_0, we have

$$L_0(X^*,\tilde{X}^*,\tilde{\tilde{V}}^*,U^*,\tilde{U}_V^*)\le L_0(X_{t+1},\tilde{X}_{t+1},\tilde{\tilde{V}}_{t+1},U^*,\tilde{U}_V^*).$$

Using $X^*=\tilde{X}^*$ and $V^*=\tilde{\tilde{V}}^*$,

$$p^*-p_{t+1}\le U^{*T}(X_{t+1}-\tilde{X}_{t+1})+\tilde{U}_V^{*T}(\tilde{\tilde{V}}_{t+1}-V_{t+1}). \tag{2}$$

By definition, X_{t+1} minimizes $L_\rho(X,\tilde{X}_t,\tilde{\tilde{V}}_t,U_t,\tilde{U}_{V_t})$. The optimality condition is

$$0\in\partial L_\rho(X_{t+1},\tilde{X}_t,\tilde{\tilde{V}}_t,U_t,\tilde{U}_{V_t}).$$

Since it is separable with respect to $(\Theta_{t+1},Z_{t+1},V_{t+1})$, we have

$$0\in\partial f(\Theta_{t+1})+U_{\Theta_t}+\rho(\Theta_{t+1}-\tilde{\Theta}_t).$$

Since $U_{\Theta_{t+1}}=U_{\Theta_t}+\rho(\Theta_{t+1}-\tilde{\Theta}_{t+1})$, we can obtain

$$0\in\partial f(\Theta_{t+1})+U_{\Theta_{t+1}}+\rho(\tilde{\Theta}_{t+1}-\tilde{\Theta}_t).$$

This implies that Θ_{t+1} minimizes

$$f(\Theta)+(U_{\Theta_{t+1}}+\rho(\tilde{\Theta}_{t+1}-\tilde{\Theta}_t))^T\Theta.$$

It follows that

$$f(\Theta_{t+1}) + (U_{\Theta_{t+1}} + \rho(\tilde{\Theta}_{t+1} - \tilde{\Theta}_t))^T \Theta_{t+1} \leq f(\Theta^*) + (U_{\Theta_{t+1}} + \rho(\tilde{\Theta}_{t+1} - \tilde{\Theta}_t))^T \Theta^*$$

Similarly, we have

$$g(Z_{t+1}) + (U_{Z_{t+1}} + \rho(\tilde{Z}_{t+1} - \tilde{Z}_t))^T Z_{t+1} \leq g(Z^*) + (U_{Z_{t+1}} + \rho(\tilde{Z}_{t+1} - \tilde{Z}_t))^T Z^*,$$

$$\begin{aligned} &h(V_{t+1}) + (U_{V_{t+1}} - \tilde{U}_{V_{t+1}} + \rho(\tilde{V}_{t+1} - \tilde{V}_t + \tilde{\tilde{V}}_{t+1} - \tilde{\tilde{V}}_t))^T V_{t+1} \\ &\leq h(V^*) + (U_{V_{t+1}} - \tilde{U}_{V_{t+1}} + \rho(\tilde{V}_{t+1} - \tilde{V}_t + \tilde{\tilde{V}}_{t+1} - \tilde{\tilde{V}}_t))^T V^* \end{aligned}$$

$$h_2(\tilde{\tilde{V}}_{t+1}) + \tilde{U}^T_{V_{t+1}} \tilde{\tilde{V}}_{t+1} \leq h_2(\tilde{\tilde{V}}^*) + \tilde{U}^T_{V_{t+1}} \tilde{\tilde{V}}^*$$

$$\Psi(\tilde{X}_{t+1}) - U^T_{t+1} \tilde{X}_{t+1} \leq \Psi(\tilde{X}^*) - U^T_{t+1} \tilde{X}^*$$

Add these inequalities above, using $X^* = \tilde{X}^*$ and $V^* = \tilde{V}^*$, we obtain

$$\begin{aligned} p_{t+1} - p^* \leq\ & U^T_{t+1}(\tilde{X}_{t+1} - X_{t+1}) + \tilde{U}^T_{V_{t+1}}(V_{t+1} - \tilde{\tilde{V}}_{t+1}) \\ & \rho(\tilde{X}_{t+1} - \tilde{X}_t)^T (X^* - X_{t+1}) + \rho(\tilde{\tilde{V}}_{t+1} - \tilde{\tilde{V}}_t)^T (V^* - V_{t+1}) \end{aligned} \tag{3}$$

Adding (2) and (3),

$$\begin{aligned} &(U_{t+1} - U^*)^T (\tilde{X}_{t+1} - X_{t+1}) + \rho(\tilde{X}_{t+1} - \tilde{X}_t)^T (X^* - X_{t+1}) \\ &+ (\tilde{U}_{V_{t+1}} - \tilde{U}^*_V)^T (V_{t+1} - \tilde{\tilde{V}}_{t+1}) + \rho(\tilde{\tilde{V}}_{t+1} - \tilde{\tilde{V}}_t)^T (V^* - V_{t+1}) \geq 0 \end{aligned} \tag{4}$$

We begin by the first two terms. Substituting $X^* - X_{t+1} = X^* - \tilde{X}_{t+1} + \tilde{X}_{t+1} - X_{t+1}$ gives

$$(U_{t+1} - U^*)^T (\tilde{X}_{t+1} - X_{t+1}) + \rho(\tilde{X}_{t+1} - \tilde{X}_t)^T (\tilde{X}_{t+1} - X_{t+1}) + \rho(\tilde{X}_{t+1} - \tilde{X}_t)^T (\tilde{X}^* - \tilde{X}_{t+1}) \tag{5}$$

For the first term of (5),

$$\begin{aligned} &(U_t + \rho(X_{t+1} - \tilde{X}_{t+1}) - U^*)^T (\tilde{X}_{t+1} - X_{t+1}) \\ &= (U_t - U^*)^T (\tilde{X}_{t+1} - X_{t+1}) - \frac{\rho}{2}\|\tilde{X}_{t+1} - X_{t+1}\|^2 - \frac{\rho}{2}\|\tilde{X}_{t+1} - X_{t+1}\|^2 \\ &= (U_t - U^*)^T (\tilde{X}_{t+1} - X_{t+1}) - \frac{\rho}{2}\|\frac{1}{\rho}(U_{t+1} - U_t)\|^2 - \frac{\rho}{2}\|\tilde{X}_{t+1} - X_{t+1}\|^2 \\ &= -\frac{1}{\rho}(U_t - U^*)^T (U_{t+1} - U^* - (U_t - U^*)) \\ &- \frac{1}{2\rho}\|U_{t+1} - U^* - (U_t - U^*)\|^2 - \frac{\rho}{2}\|\tilde{X}_{t+1} - X_{t+1}\|^2 \\ &= \frac{1}{2\rho}\|U_t - U^*\|^2 - \frac{1}{2\rho}\|U_{t+1} - U^*\|^2 - \frac{\rho}{2}\|\tilde{X}_{t+1} - X_{t+1}\|^2. \end{aligned}$$

For the last two terms of (5), taking $\frac{\rho}{2}\|\tilde{X}_{t+1} - X_{t+1}\|^2$ from above,

$$
\begin{aligned}
&-\frac{\rho}{2}\|\tilde{X}_{t+1} - X_{t+1}\|^2 + \rho(\tilde{X}_{t+1} - \tilde{X}_t)^T(\tilde{X}_{t+1} - X_{t+1}) \\
&+ \rho(\tilde{X}_{t+1} - \tilde{X}_t)^T(\tilde{X}^* - \tilde{X}_t + \tilde{X}_t - \tilde{X}_{t+1}) \\
&= -\frac{\rho}{2}\|\tilde{X}_{t+1} - X_{t+1} - (\tilde{X}_{t+1} - \tilde{X}_t)\|^2 + \rho(\tilde{X}_{t+1} - \tilde{X}_t)(\tilde{X}^* - \tilde{X}_t) - \frac{\rho}{2}\|\tilde{X}_{t+1} - \tilde{X}_t\|^2 \\
&= -\frac{\rho}{2}\|\tilde{X}_{t+1} - X_{t+1} - (\tilde{X}_{t+1} - \tilde{X}_t)\|^2 - \frac{\rho}{2}\|\tilde{X}_{t+1} - \tilde{X}^*\|^2 + \frac{\rho}{2}\|\tilde{X}_t - \tilde{X}^*\|^2
\end{aligned}
$$

Hence the first two terms of (4) can be rewritten as

$$\frac{1}{2\rho}\|U_t - U^*\|^2 - \frac{1}{2\rho}\|U_{t+1} - U^*\|^2 - \frac{\rho}{2}\|\tilde{X}_{t+1} - X_{t+1} - (\tilde{X}_{t+1} - \tilde{X}_t)\|^2 + \frac{\rho}{2}\|\tilde{X}_t - \tilde{X}^*\|^2 - \frac{\rho}{2}\|\tilde{X}_{t+1} - \tilde{X}^*\|^2$$

Similarly, the last two terms of (4) can be rewritten as

$$\frac{1}{2\rho}\|\tilde{U}_{V_t} - \tilde{U}_V^*\|^2 - \frac{1}{2\rho}\|\tilde{U}_{V_{t+1}} - \tilde{U}_V^*\|^2 - \frac{\rho}{2}\|\tilde{\tilde{V}}_{t+1} - V_{t+1} - (\tilde{\tilde{V}}_{t+1} - \tilde{\tilde{V}}_t)\|^2 + \frac{\rho}{2}\|V_t - \tilde{\tilde{V}}^*\|^2 - \frac{\rho}{2}\|\tilde{\tilde{V}}_{t+1} - \tilde{\tilde{V}}^*\|^2$$

This implies that (4) can be written as

$$
\begin{aligned}
&\frac{1}{2\rho}\|U_t - U^*\|^2 + \frac{1}{2\rho}\|\tilde{U}_{V_t} - \tilde{U}_V^*\|^2 + \frac{\rho}{2}\|\tilde{X}_t - \tilde{X}^*\|^2 + \frac{\rho}{2}\|V_t - \tilde{\tilde{V}}^*\|^2 \\
&- \frac{1}{2\rho}\|U_{t+1} - U^*\|^2 - \frac{1}{2\rho}\|\tilde{U}_{V_{t+1}} - \tilde{U}_V^*\|^2 - \frac{\rho}{2}\|\tilde{X}_{t+1} - \tilde{X}^*\|^2 - \frac{\rho}{2}\|\tilde{\tilde{V}}_{t+1} - \tilde{\tilde{V}}^*\|^2 \\
&- \frac{\rho}{2}\|\tilde{X}_{t+1} - X_{t+1} - (\tilde{X}_{t+1} - \tilde{X}_t)\|^2 - \frac{\rho}{2}\|\tilde{\tilde{V}}_{t+1} - V_{t+1} - (\tilde{\tilde{V}}_{t+1} - \tilde{\tilde{V}}_t)\|^2 \geq 0.
\end{aligned}
\tag{6}
$$

Let

$$D_t = \frac{1}{2\rho}\|U_t - U^*\|^2 + \frac{1}{2\rho}\|\tilde{U}_{V_t} - \tilde{U}_V^*\|^2 + \frac{\rho}{2}\|\tilde{X}_t - \tilde{X}^*\|^2 + \frac{\rho}{2}\|V_t - \tilde{\tilde{V}}^*\|^2$$

it follows that

$$D_t - D_{t+1} \geq \frac{\rho}{2}\|\tilde{X}_{t+1} - X_{t+1} - (\tilde{X}_{t+1} - \tilde{X}_t)\|^2 + \frac{\rho}{2}\|\tilde{\tilde{V}}_{t+1} - V_{t+1} - (\tilde{\tilde{V}}_{t+1} - \tilde{\tilde{V}}_t)\|^2 \tag{7}$$

Recall that $\tilde{X}_{t+1}$ minimizes $\Psi(\tilde{X}) - U_{t+1}^T\tilde{X}$ and $\tilde{X}_t$ minimizes $\Psi(\tilde{X}) - U_t^T\tilde{X}$, so we can add

$$\Psi(\tilde{X}_{t+1}) - U_{t+1}^T\tilde{X}_{t+1} \leq \Psi(\tilde{X}_t) - U_{t+1}^T\tilde{X}_t$$

and

$$\Psi(\tilde{X}_t) - U_t^T\tilde{X}_t \leq \Psi(\tilde{X}_{t+1}) - U_t^T\tilde{X}_{t+1}$$

to get

$$(U_{t+1} - U_t)^T(\tilde{X}_{t+1} - \tilde{X}_t) \geq 0.$$

Hence by $\rho > 0$,

$$(\tilde{X}_{t+1} - X_{t+1})^T(\tilde{X}_{t+1} - \tilde{X}_t) \leq 0.$$

Likewise,

$$(\tilde{\tilde{V}}_{t+1} - V_{t+1})^T(\tilde{\tilde{V}}_{t+1} - \tilde{\tilde{V}}_t) \leq 0.$$

Combined with (7),

$$D_t - D_{t+1} \geq \frac{\rho}{2}\|\tilde{X}_{t+1} - X_{t+1}\|^2 + \frac{\rho}{2}\|\tilde{X}_{t+1} - \tilde{X}_t\|^2 + \frac{\rho}{2}\|\tilde{\tilde{V}}_{t+1} - V_{t+1}\|^2 + \frac{\rho}{2}\|\tilde{\tilde{V}}_{t+1} - \tilde{\tilde{V}}_t\|^2.$$

Iterating these inequalities gives that

$$\sum_{t=0}^{\infty} \frac{\rho}{2}\|\tilde{X}_{t+1} - X_{t+1}\|^2 + \frac{\rho}{2}\|\tilde{X}_{t+1} - \tilde{X}_t\|^2 + \frac{\rho}{2}\|\tilde{\tilde{V}}_{t+1} - V_{t+1}\|^2 + \frac{\rho}{2}\|\tilde{\tilde{V}}_{t+1} - \tilde{\tilde{V}}_t\|^2 \leq D_0$$

which implies that $\tilde{X}_{t+1} - X_{t+1} \to 0$ and $\tilde{\tilde{V}}_{t+1} - V_{t+1} \to 0$. The right hand in (2) goes to zero as $t \to \infty$. Hence we have $\lim_{t\to\infty} p_t = p^*$.

Reference

[1] Stephen Boyd, Stephen P Boyd, and Lieven Vandenberghe. *Convex optimization.* Cambridge university press, 2004.
