# Supplementary material for: Extended graphical lasso for multiple interaction networks for high dimensional omics data
Source: PLoS Comput Biol. 2021 Oct 20;17(10):e1008794. doi: 10.1371/journal.pcbi.1008794 (PMC8528283; doi:10.1371/journal.pcbi.1008794)
Supplement: S3 Text — (PDF) [file pcbi.1008794.s003.pdf]

S3 Text: The proof of the sufficient condition for the non-uniform block diagonal structure

Let $\Omega_k = \bigcup_{t=1}^{T_k}\{C_t^k * C_t^k\}, S_{\max}^{(k)} = \max_{(i,j)\in\Omega_k^c}|S_{ij}^{(k)}|$, let A_{Ω_k} denote the restriction of the matrix A to the set Ω_k, that is

$$(A_{\Omega_k})_{ij} = \begin{cases} A_{ij}, & \text{if } (i,j) \in \Omega_k; \\ 0, & \text{if } (i,j) \notin \Omega_k. \end{cases}$$

Assume $(\{\Theta^{(k)}\}, \{Z^{(k)}\}, \{V^{(k)}\})$ is a feasible solution, then $(\{\Theta_{\Omega_k}^{(k)}\}, \{Z_{\Omega_k}^{(k)}\}, \{V_{\Omega_k}^{(k)}\})$ is also a feasible solution. We want to show that if the condition holds, then the objective value evaluated at $(\{\Theta_{\Omega_k}^{(k)}\}, \{Z_{\Omega_k}^{(k)}\}, \{V_{\Omega_k}^{(k)}\})$ is smaller than the objective value evaluated at $(\{\Theta^{(k)}\}, \{Z^{(k)}\}, \{V^{(k)}\})$. By Fischer's inequality,

$$-\text{logdet}(\Theta^{(k)}) > -\text{logdet}(\Theta_{\Omega_k}^{(k)})$$

We need only to prove

$$\begin{aligned}
&\sum_{k=1}^{K} n_k \text{tr}(S^{(k)}\Theta^{(k)}) + \lambda_1 \sum_{k=1}^{K} ||Z^{(k)} - \text{diag}(Z^{(k)})||_1 + \lambda_3 \sum_{k=1}^{K} ||V^{(k)} - \text{diag}(V^{(k)})||_1 \\
&+ \lambda_2 \sum_{k<k'} ||Z^{(k)} - Z^{(k')} - \text{diag}(Z^{(k)} - Z^{(k')})||_1 + \lambda_4 \sum_{k=1}^{K} ||V^{(k)} - \text{diag}(V^{(k)})||_{1,2} \\
&+ \lambda_5 \sum_{k<k'} ||V^{(k)} - V^{(k')} - \text{diag}(V^{(k)} - V^{(k')})||_1 \\
&\geq \sum_{k=1}^{K} n_k \text{tr}(S^{(k)}\Theta_{\Omega_k}^{(k)}) + \lambda_1 \sum_{k=1}^{K} ||Z_{\Omega_k}^{(k)} - \text{diag}(Z_{\Omega_k}^{(k)})||_1 + \lambda_3 \sum_{k=1}^{K} ||V_{\Omega_k}^{(k)} - \text{diag}(V_{\Omega_k}^{(k)})||_1 \\
&+ \lambda_2 \sum_{k<k'} ||Z_{\Omega_k}^{(k)} - Z_{\Omega_{k'}}^{(k')} - \text{diag}(Z_{\Omega_k}^{(k)} - Z_{\Omega_{k'}}^{(k')})||_1 + \lambda_4 \sum_{k=1}^{K} ||V_{\Omega_k}^{(k)} - \text{diag}(V_{\Omega_k}^{(k)})||_{1,2} \\
&+ \lambda_5 \sum_{k<k'} ||V_{\Omega_k}^{(k)} - V_{\Omega_{k'}}^{(k')} - \text{diag}(V_{\Omega_k}^{(k)} - V_{\Omega_{k'}}^{(k')})||_1
\end{aligned} \tag{1}$$

As

$$\begin{aligned}
&\sum_{k<k'} ||Z^{(k)} - Z^{(k')}||_1 - \sum_{k<k'} ||Z_{\Omega_k}^{(k)} - Z_{\Omega_{k'}}^{(k')}||_1 \\
&= ||Z_{\Omega_k^c \bigcup \Omega_{k'}^c}^{(k)} - Z_{\Omega_k^c \bigcup \Omega_{k'}^c}^{(k')}||_1 - ||Z_{\Omega_k \bigcap \Omega_{k'}^c}^{(k)}||_1 - ||Z_{\Omega_k^c \bigcap \Omega_{k'}}^{(k')}||_1,
\end{aligned}$$

$$||V^{(k)}||_{1,2} \geq ||V^{(k)}_{\Omega_k}||_{1,2},$$

$$\sum_{k<k'} ||V^{(k)} - V^{(k')}||_1 - \sum_{k<k'} ||V^{(k)}_{\Omega_k} - V^{(k')}_{\Omega_{k'}}||_1$$

$$= ||V^{(k)}_{\Omega_k^c \bigcup \Omega_{k'}^c} - V^{(k')}_{\Omega_k^c \bigcup \Omega_{k'}^c}||_1 - ||V^{(k)}_{\Omega_k \bigcap \Omega_{k'}^c}||_1 - ||V^{(k')}_{\Omega_k^c \bigcap \Omega_{k'}}||_1,$$

Hence we only need to prove

$$\sum_{k=1}^{K} n_k < S^{(k)}_{\Omega_k^c}, \Theta^{(k)}_{\Omega_k^c} > +\lambda_1 \sum_{k=1}^{K} ||Z^{(k)}_{\Omega_k^c}||_1$$

$$+ \lambda_2 \sum_{k<k'} (||Z^{(k)}_{\Omega_k^c \bigcup \Omega_{k'}^c} - Z^{(k')}_{\Omega_k^c \bigcup \Omega_{k'}^c}||_1 - ||Z^{(k)}_{\Omega_k \bigcap \Omega_{k'}^c}||_1 - ||Z^{(k')}_{\Omega_k^c \bigcap \Omega_{k'}}||_1)$$

$$+ \lambda_3 \sum_{k=1}^{K} ||V^{(k)}_{\Omega_k^c}||_1 + \lambda_5 \sum_{k<k'} (||V^{(k)}_{\Omega_k^c \bigcup \Omega_{k'}^c} - V^{(k')}_{\Omega_k^c \bigcup \Omega_{k'}^c}||_1 - ||V^{(k)}_{\Omega_k \bigcap \Omega_{k'}^c}||_1 - ||V^{(k')}_{\Omega_k^c \bigcap \Omega_{k'}}||_1) \geq 0$$

As $||Z^{(k)}_{\Omega_k \bigcap \Omega_{k'}^c}||_1 \leq ||Z^{(k)}_{\Omega_k}||_1$ and $||V^{(k)}_{\Omega_k \bigcap \Omega_{k'}^c}||_1 \leq ||V^{(k)}_{\Omega_k}||_1$, we need to prove that

$$\sum_{k=1}^{K} n_k < S^{(k)}_{\Omega_k^c}, \Theta^{(k)}_{\Omega_k^c} > +(\lambda_1 - (K-1)\lambda_2) \sum_{k=1}^{K} ||Z^{(k)}_{\Omega_k^c}||_1 + (\lambda_3 - (K-1)\lambda_5) \sum_{k=1}^{K} ||V^{(k)}_{\Omega_k^c}||_1 \geq 0$$

$$\sum_{k=1}^{K} n_k | < S^{(k)}_{\Omega_k^c}, \Theta^{(k)}_{\Omega_k^c} > |$$

$$= \sum_{k=1}^{K} n_k | < S^{(k)}_{\Omega_k^c}, Z^{(k)}_{\Omega_k^c} + V^{(k)}_{\Omega_k^c} + (V^{(k)}_{\Omega_k^c})^T > |$$

$$\leq \sum_{k=1}^{K} n_k | < S^{(k)}_{\Omega_k^c}, Z^{(k)}_{\Omega_k^c} > | + 2 \sum_{k=1}^{K} n_k | < S^{(k)}_{\Omega_k^c}, V^{(k)}_{\Omega_k^c} > |$$

$$\leq \sum_{k=1}^{K} n_k S^{(k)}_{\max} ||Z^{(k)}_{\Omega_k^c}||_1 + 2 \sum_{k=1}^{K} n_k S^{(k)}_{\max} ||V^{(k)}_{\Omega_k^c}||_1$$

$$\leq (\lambda_1 + \lambda_2 - K\lambda_2) \sum_{k=1}^{K} ||Z^{(k)}_{\Omega_k^c}||_1 + (\lambda_3 + \lambda_5 - K\lambda_5) \sum_{k=1}^{K} ||V^{(k)}_{\Omega_k^c}||_1$$

where the last inequality follows from the sufficient condition.
