# Supplementary material for: Extended graphical lasso for multiple interaction networks for high dimensional omics data
Source: PLoS Comput Biol. 2021 Oct 20;17(10):e1008794. doi: 10.1371/journal.pcbi.1008794 (PMC8528283; doi:10.1371/journal.pcbi.1008794)
Supplement: S4 Text — (PDF) [file pcbi.1008794.s004.pdf]

## S4 Text: The proof of Theorem 3

Let $(\Theta^{*(k)}, Z^{*(k)}, V^{*(k)})$ be the solution to problem and suppose that $Z^{*(k)}$ is not a diagonal matrix. Let $\hat{Z}^{(k)} = \text{diag}(Z^{*(k)})$, and construct $\hat{V}$ as follows

$$\hat{V}_{ij}^{(k)} = \begin{cases} V_{ij}^{*(k)} + \frac{Z_{ij}^{*(k)}}{2} & \text{if} \quad i \neq j \\ V_{ij}^{*(k)} & \text{otherwise} \end{cases}$$

Then we have $\Theta^{*(k)} = \hat{Z}^{(k)} + \hat{V}^{(k)} + t(\hat{V}^{(k)})$. Thus $(\Theta^{*(k)}, \hat{Z}^{(k)}, \hat{V}^{(k)})$ is also a feasible solution. Now we prove that if the condition holds, $(\Theta^{*(k)}, \hat{Z}^{(k)}, \hat{V}^{(k)})$ has a smaller objective than $(\Theta^{*(k)}, Z^{*(k)}, V^{*(k)})$. We need to prove that

$$\begin{aligned} &\lambda_3 \sum_{k=1}^{K} ||\hat{V}^{(k)} - \text{diag}(\hat{V}^{(k)})||_1 + \lambda_4 \sum_{k=1}^{K} ||\hat{V}^{(k)} - \text{diag}(\hat{V}^{(k)})||_{1,2} \\ &+ \lambda_5 \sum_{k<k'} ||\hat{V}^{(k)} - \hat{V}^{(k')} - \text{diag}(\hat{V}^{(k)} - \hat{V}^{(k')})||_1 \\ &\leq \lambda_1 \sum_{k=1}^{K} ||Z^{*(k)} - \text{diag}(Z^{*(k)})||_1 \\ &+ \lambda_2 \sum_{k<k'} ||Z^{*(k)} - Z^{*(k')} - \text{diag}(Z^{*(k)} - Z^{*(k')})||_1 \\ &+ \lambda_3 \sum_{k=1}^{K} ||V^{*(k)} - \text{diag}(V^{*(k)})||_1 + \lambda_4 \sum_{k=1}^{K} ||V^{*(k)} - \text{diag}(V^{*(k)})||_{1,2} \\ &+ \lambda_5 \sum_{k<k'} ||V^{*(k)} - V^{*(k')} - \text{diag}(V^{*(k)} - V^{*(k')})||_1. \end{aligned} \tag{1}$$

Three terms in the left hand of formula (1) can be restricted separately.

$$\begin{aligned} &\lambda_3 \sum_{k=1}^{K} ||\hat{V}^{(k)} - \text{diag}(\hat{V}^{(k)})||_1 \\ &\leq \lambda_3 \sum_{k=1}^{K} ||V^{*(k)} - \text{diag}(V^{*(k)})||_1 + \frac{\lambda_3}{2} \sum_{k=1}^{K} ||Z^{*(k)} - \text{diag}(Z^{*(k)})||_1. \end{aligned}$$

$$\begin{aligned} &\lambda_4 \sum_{k=1}^{K} ||\hat{V}^{(k)} - \text{diag}(\hat{V}^{(k)})||_{1,2} \\ &\leq \lambda_4 \sum_{k=1}^{K} ||V^{*(k)} - \text{diag}(V^{*(k)})||_{1,2} + \frac{\lambda_4}{2} \sum_{k=1}^{K} ||Z^{*(k)} - \text{diag}(Z^{*(k)})||_{1,2}. \end{aligned}$$

$$
\begin{aligned}
&\lambda_5 \sum_{k<k'} ||\hat{V}^{(k)} - \hat{V}^{(k')} - \text{diag}(\hat{V}^{(k)} - \hat{V}^{(k')})||_1 \\
&\leq \lambda_5 \sum_{k<k'} ||V^{*(k)} - V^{*(k')} - \text{diag}(V^{*(k)} - V^{*(k')})||_1 \\
&+ \frac{\lambda_5}{2} \sum_{k<k'} ||Z^{*(k)} - Z^{*(k')} - \text{diag}(Z^{*(k)} - Z^{*(k')})||_1.
\end{aligned}
$$

Then the inequality (1) follows from the sufficient condition.
