# Supplementary material for: Extended graphical lasso for multiple interaction networks for high dimensional omics data
Source: PLoS Comput Biol. 2021 Oct 20;17(10):e1008794. doi: 10.1371/journal.pcbi.1008794 (PMC8528283; doi:10.1371/journal.pcbi.1008794)
Supplement: S5 Text — (PDF) [file pcbi.1008794.s005.pdf]

S5 Text: The proof of Theorem 4

Let $(\Theta^{*(k)}, Z^{*(k)}, V^{*(k)})$ be the solution to problem and suppose that $V^{*(k)}$ is not a diagonal matrix. Let $\hat{V}^{(k)} = \mathrm{diag}(V^{*(k)})$, and construct $\hat{Z}$ as follows

$$\hat{Z}_{ij}^{(k)} = \begin{cases} Z_{ij}^{*(k)} + V_{ij}^{*(k)} + V_{ji}^{*(k)} & \text{if} \quad i \neq j \\ Z_{ij}^{*(k)} & \text{otherwise} \end{cases}$$

Then we have $\Theta^{*(k)} = \hat{Z}^{(k)} + \hat{V}^{(k)} + t(\hat{V}^{(k)})$. Thus $(\Theta^{*(k)}, \hat{Z}^{(k)}, \hat{V}^{(k)})$ is also a feasible solution. Now we prove that if the condition holds, $(\Theta^{*(k)}, \hat{Z}^{(k)}, \hat{V}^{(k)})$ has a smaller objective than $(\Theta^{*(k)}, Z^{*(k)}, V^{*(k)})$. We only need to prove that

$$\begin{aligned} &\lambda_1 \sum_{k=1}^{k} ||\hat{Z}^{(k)} - \mathrm{diag}(\hat{Z}^{(k)})||_1 \\ &+ \lambda_2 \sum_{k<k'} ||\hat{Z}^{(k)} - \hat{Z}^{(k')} - \mathrm{diag}(\hat{Z}^{(k)} - \hat{Z}^{(k')})||_1 \\ \leq\ &\lambda_1 \sum_{k=1}^{k} ||Z^{*(k)} - \mathrm{diag}(Z^{*(k)})||_1 + \\ &+ \lambda_2 \sum_{k<k'} ||Z^{*(k)} - Z^{*(k')} - \mathrm{diag}(Z^{*(k)} - Z^{*(k')})||_1 \\ &+ \lambda_3 \sum_{k=1}^{K} ||V^{*(k)} - \mathrm{diag}(V^{*(k)})||_1 + \lambda_4 \sum_{k=1}^{K} ||V^{*(k)} - \mathrm{diag}(V^{*(k)})||_{1,2} \\ &+ \lambda_5 \sum_{k<k'} ||V^{*(k)} - V^{*(k')} - \mathrm{diag}(V^{*(k)} - V^{*(k')})||_1. \end{aligned} \tag{1}$$

Two terms in the left hand of formula (1) can be restricted separately.

$$\begin{aligned} &\lambda_1 \sum_{k=1}^{k} ||\hat{Z}^{(k)} - \mathrm{diag}(\hat{Z}^{(k)})||_1 \\ \leq\ &\lambda_1 \sum_{k=1}^{k} ||Z^{*(k)} - diag(Z^{*(k)})||_1 + 2\lambda_1 \sum_{k=1}^{K} ||V^{*(k)} - diag(V^{*(k)})||_1. \end{aligned}$$

$$\begin{aligned} &\lambda_2 \sum_{k<k'} ||\hat{Z}^{(k)} - \hat{Z}^{(k')} - \mathrm{diag}(\hat{Z}^{(k)} - \hat{Z}^{(k')})||_1 \\ \leq \quad &\lambda_2 \sum_{k<k'} ||Z^{*(k)} - Z^{*(k')} - diag(Z^{*(k)} - Z^{*(k')})||_1 \\ &+ 2\lambda_2 \sum_{k<k'} ||V^{*(k)} - V^{*(k')} - diag(V^{*(k)} - V^{*(k')})||_1. \end{aligned}$$

And

$$\sum_{k=1}^{K} ||V^{*(k)} - diag(V^{*(k)})||_1 \leq \sqrt{p} \sum_{k=1}^{K} ||V^{*(k)} - \text{diag}(V^{*(k)})||_{1,2},$$

by Cauchy inequality.

Then the inequality (1) follows from the sufficient condition.
