# Supplementary material for: Extended graphical lasso for multiple interaction networks for high dimensional omics data
Source: PLoS Comput Biol. 2021 Oct 20;17(10):e1008794. doi: 10.1371/journal.pcbi.1008794 (PMC8528283; doi:10.1371/journal.pcbi.1008794)
Supplement: S6 Text — (PDF) [file pcbi.1008794.s006.pdf]

S6 Text: Methods for generating the basis proportion for each feature

We generate count data according to two steps. (1) In the first step, we sample basis proportions for each OTU given mean basis abundance and basis covariance, from one of three underlying distributions, such as log ratio normal (LRN), Poisson log normal (LNP), and Dirichlet log normal (LND). (2) For each sample, counts are drawn from Multinomial distribution using the basis proportions obtained in the first step with a sample size. Suppose that basis abundance $a = (a_1, a_2, \ldots, a_p)^T$ has basis covariance Σ_{p*p}. For L-RN, we sample $\phi_i = \log\left(\frac{x_i}{x_p}\right) \sim MVN(\mu, \Omega)$ for $i = 1, 2, \ldots, p-1$, where $\mu = \log\left(\frac{(a_1, a_2, \ldots, a_{p-1})^T}{a_p}\right)$, $\Omega = L\Sigma L^T$,and

$$L = \begin{pmatrix} 1 & \cdots & 0 & -1 \\ \vdots & \ddots & \vdots & \vdots \\ 0 & \cdots & 1 & -1 \end{pmatrix}_{(p-1)*p}$$

Then, we can obtain basis proportions $w_i = \frac{exp(\phi_i)}{1+\sum_{i=1}^{p-1} exp(\phi_i)}$ for $i = 1, \ldots, p-1$ and $w_p = \frac{1}{1+\sum_{i=1}^{p-1} exp(\phi_i)}$. For LNP, we sample ϕ_i such that $\log(\phi_i) \sim MVN(0, \Sigma)$. Then we obtain for each OTU basis abundance $c_i|\phi_i \sim Poisson(a_i\phi_i)$ and proportions $w_i = \frac{c_i}{\sum_{i=1}^{p} c_i}$. For LND, we sample ϕ_i such that $\log(\phi_i) \sim MVN(0, \Sigma)$. Then for each sample we draw the basis proportions $w_1, \ldots, w_p|\phi \sim \frac{\Gamma(\sum_{i=1}^{p} \phi_i)}{\prod_{i=1}^{p} \Gamma(\phi_i)} \prod_{i=1}^{p} w_i^{\phi_i - 1}$.

In the second step, the count data are drawn from $x_1, x_2, \ldots, x_p \sim C_X^x \prod_{i=1}^{p} w_i^{x_i}$ for each sample given a sequencing size of $X = \sum_{i=1}^{p} x_i$.
