# Supplementary material for: Extended graphical lasso for multiple interaction networks for high dimensional omics data
Source: PLoS Comput Biol. 2021 Oct 20;17(10):e1008794. doi: 10.1371/journal.pcbi.1008794 (PMC8528283; doi:10.1371/journal.pcbi.1008794)
Supplement: S7 Text — (PDF) [file pcbi.1008794.s007.pdf]

S7 Text: A simple example computation of TPR and FPR based on current method

Below we explain how we compute the TPR (True Positive Rate), FPR (False Positive Rate) and Precision with an example.

Suppose the nodes are labelled as $1, 2., ..., p$ and our program detects the following nodes as hubs under three conditions $\langle 1 \rangle, \langle 2 \rangle, \langle 3 \rangle$:

```
<1> 7, 15, 22, 36, 60, 77, 79, 84, 145, 154
<2> 15, 22, 60, 77, 79, 98, 145, 154
<3> 12, 15, 22, 60, 67, 100, 109, 135, 145, 154, 159
```

Suppose the true hubs are located at these nodes under three conditions $\langle 1 \rangle, \langle 2 \rangle, \langle 3 \rangle$:

```
<1> 7, 15, 22, 36, 60, 79, 84, 154
<2> 15, 36, 60, 77, 79, 98, 145, 154
<3> 15, 22, 60, 100, 109, 145, 154, 159
```

When the relevant elements to detect are the (true) common hubs 15, 60, 154, our program successfully detects node 15, 60, 154, so

$$\text{TPR-C} = \frac{TP}{P} = 1$$

Out of the $p - 3$ node locations which are not true common hubs, our program falsely detects node 22, 145 as common hubs, so

$$\text{FPR-C} = \frac{FP}{N} = \frac{2}{p-3}$$

$$\text{Precision-C} = \frac{TP}{TP + FP} = \frac{3}{3+2}$$

When the relevant elements to detect are the eleven (true) conditional-specific hubs 36,79 (under $\langle 1, 2 \rangle$), 145 (under $\langle 2, 3 \rangle$), and 22 (under $\langle 1, 3 \rangle$), 7, 84 (under $\langle 1 \rangle$), 77, 98 (under $\langle 2 \rangle$), 100, 109, 159 (under $\langle 3 \rangle$), our program successfully detects nodes 7, 79, 84, 98, 100, 109, 159 so

$$\text{TPR-S} = \frac{TP}{P} = \frac{7}{11}$$

Out of the $p-11$ nodes which are not conditional-specific hubs (including all nodes which are either true non-hubs or true common hubs), our program makes one mistake: it falsely detects true common hub 12, 67, 135 as condition $\langle 3 \rangle$-specific. So

$$\text{FPR-S} = \frac{FP}{N} = \frac{3}{p-11}$$

$$\text{Precision-S} = \frac{TP}{TP+FP} = \frac{7}{7+3}.$$

Overall, the number of true hubs for each class is eight, and we successfully detect 8, 7 (missing 36), 8 respectively. Out of the $3p-24$ nodes we falsely identify 77, 145 (under $\langle 1 \rangle$), 22 (under $\langle 2 \rangle$), 12, 67, 135 (under $\langle 3 \rangle$). The corresponding total TPR, FPR and Precision are compute as

$$\text{TPR} = \frac{8+7+8}{8+8+8}$$

$$\text{FPR} = \frac{2+1+3}{3p-24}$$

$$\text{Precision} = \frac{8+7+8}{10+8+11}.$$
