# Supplementary material for: Extended graphical lasso for multiple interaction networks for high dimensional omics data
Source: PLoS Comput Biol. 2021 Oct 20;17(10):e1008794. doi: 10.1371/journal.pcbi.1008794 (PMC8528283; doi:10.1371/journal.pcbi.1008794)
Supplement: S1 Table — The ability of EDOHA, JRmGRN and HGL to identify hub nodes in the networks with “only common hubs” and “only class-specific hubs” are shown by True Positive Rate (TPR) and False Positive Rate (FPR). We generate 100 datasets with sample size n = 80. (PDF) [file pcbi.1008794.s008.pdf]

S1 Table: Additional simulations for only common hubs and only class-specific hubs

Table A: The ability of EDOHA, JRmGRN and HGL to identify hub nodes in the networks with "only common hubs" are shown by True Positive Rate (TPR) and False Positive Rate (FPR). We generate 100 datasets with sample size n=80.

	80 nodes: 5 hubs		160 nodes: 8 hubs		300 nodes: 12 hubs	
	TPR	FPR	TPR	FPR	TPR	FPR
EDOHA	0.821	0.013	0.796	0.003	0.763	0.001
JRmGRN	0.895	0.017	0.917	0.016	0.875	0.004
HGL	0.753	0.024	0.769	0.002	0.662	0.001

Table B: The ability of EDOHA, JRmGRN and HGL to identify hub nodes in the networks with "only class-specific hubs" are shown by True Positive Rate (TPR) and False Positive Rate (FPR). We generate 100 datasets with sample size n=80.

	80 nodes: 5 hubs		160 nodes: 8 hubs		300 nodes: 12 hubs	
	TPR	FPR	TPR	FPR	TPR	FPR
EDOHA	0.871	0.027	0.746	0.026	0.711	0.025
HGL	0.879	0.046	0.797	0.021	0.715	0.01
JRmGRN	NA	NA	NA	NA	NA	NA
