# Supplementary material for: Extended graphical lasso for multiple interaction networks for high dimensional omics data
Source: PLoS Comput Biol. 2021 Oct 20;17(10):e1008794. doi: 10.1371/journal.pcbi.1008794 (PMC8528283; doi:10.1371/journal.pcbi.1008794)
Supplement: S2 Table — The table lists hub proteins detected as common ones as well as tissue-specific ones, and introduces their functions. (PDF) [file pcbi.1008794.s009.pdf]

S2 Table: The hub proteins detected in four organs and their functions in living organism

Hub	Tissue	Function
DDX21	Colon; Liver; Lung; Kidney	play an important role in ribosomal RNA biogenesis, RNA editing, RNA transport, and general transcription
REEP6	Colon; Liver; Lung; Kidney	the transport of receptors from the endoplasmic reticulum (ER) to the cell surface and regulates ER membrane structure
SEPSECS	Colon; Liver; Lung; Kidney	convert O-phosphoseryl-tRNA(Sec) to selenocysteinyl-tRNA(Sec) required for selenoprotein biosynthesis
TIMM9	Liver; Lung; Kidney	mediate the import and insertion of hydrophobic membrane proteins into the mitochondrial inner membrane
HMOX1	Liver; Kidney	an essential enzyme in heme catabolism
PRKAR2B	Colon; Liver	regulatory subunit of the cAMP-dependent protein kinases involved in cAMP signaling in cells, and cAMP is a signaling molecule important for a variety of cellular functions
MRPS5	Colon	RNA binding and structural constituent of ribosome
BCKDK	Liver	the key regulatory enzyme of the valine, leucine and isoleucine catabolic pathways and regulates the activity state of the BCKD complex
COMT	Liver	participate in the metabolism of endogenous substances
BZW2	Lung	be involved in cell differentiation and nervous system development
SLC44A2	Lung	exhibit some choline transporter activity
STOM	Lung	regulate ion channel activity and transmembrane ion transport

ATP1B1	Kidney	the non-catalytic component of the active enzyme, which catalyzes the hydrolysis of ATP coupled with the exchange of Na^+ and K^+ ions across the plasma membrane
ATP6AP1	Kidney	be required for luminal acidification of secretory vesicles.
ATP6V1A	Kidney	mediate acidification of eukaryotic intracellular organelles and play a part in neurite development and synaptic connectivity
CCDC86	Kidney	be involved in RNA binding and viral process
ETFA	Kidney	be required for normal mitochondrial fatty acid oxidation snd normal amino acid metabolism
NUP210	Kidney	a membrane-spanning glycoprotein that is a major component of the nuclear pore complex
PTGES2	Kidney	Isomerase that catalyzes the conversion of PGH2 into the more stable prostaglandin E2 (PGE2)
SCARB1	Kidney	a plasma membrane receptor for high density lipoprotein cholesterol (HDL) mediate cholesterol transfer to and from HDL
