# Supplementary figures and images for: Extended graphical lasso for multiple interaction networks for high dimensional omics data
Source: PLoS Comput Biol. 2021 Oct 20;17(10):e1008794. doi: 10.1371/journal.pcbi.1008794 (PMC8528283; doi:10.1371/journal.pcbi.1008794)

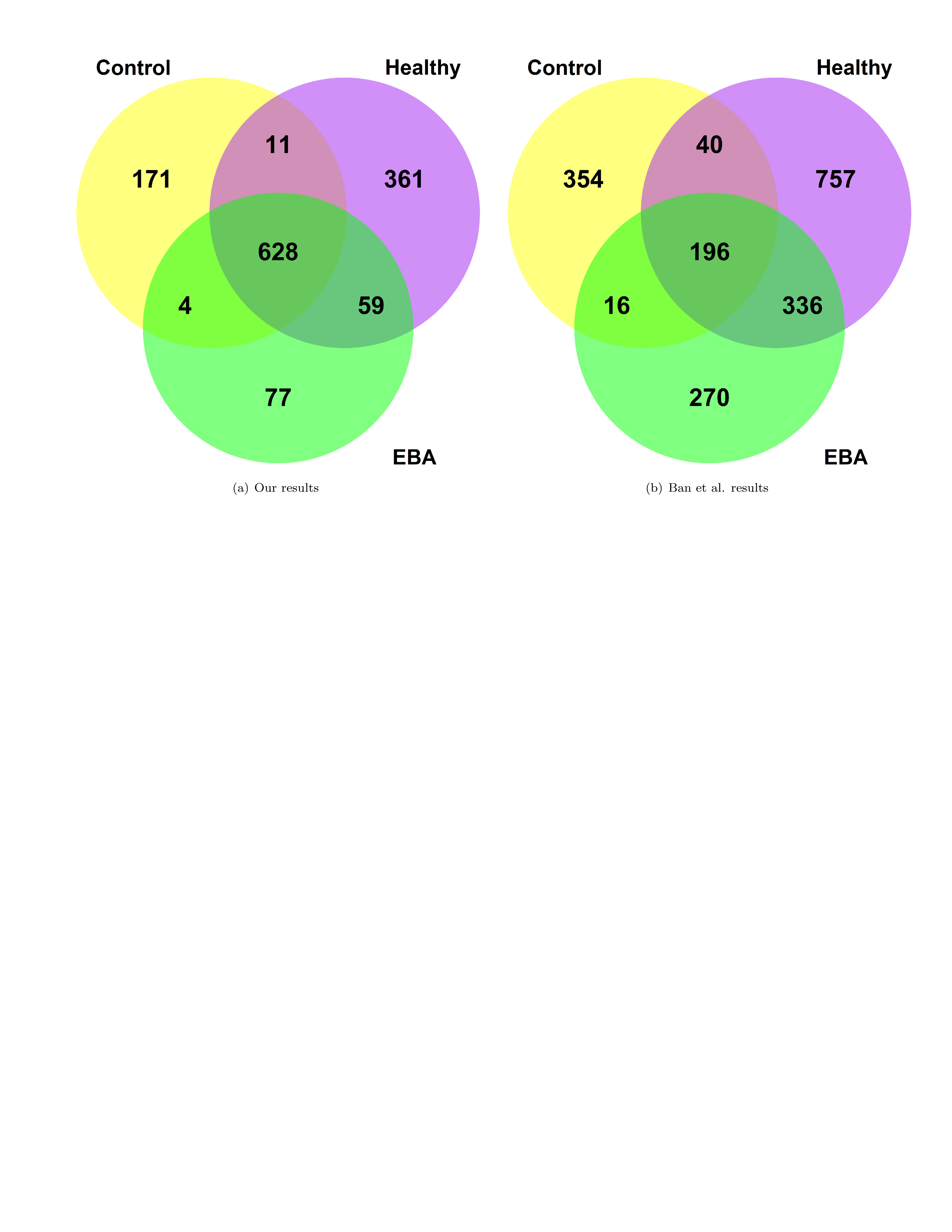

Supplement: S1 Fig — The figure shows the number of possible pairs within the same group and between different groups. It is suggested that the gaps between these groups in our research are much less than Ban et al. [21]. (TIF) [file pcbi.1008794.s010.tif]
